# Fibronectin Fragments and Inflammation During Canine Intervertebral Disc Disease

**DOI:** 10.3389/fvets.2020.547644

**Published:** 2020-11-16

**Authors:** Manuel Roland Schmidli, Aleksandra Sadowska, Iva Cvitas, Benjamin Gantenbein, Heidi E. L. Lischer, Simone Forterre, Wolfgang Hitzl, Franck Forterre, Karin Wuertz-Kozak

**Affiliations:** ^1^Division of Small Animal Surgery and Orthopaedics, Department of Clinical Veterinary Medicine, Vetsuisse Faculty, University of Bern, Bern, Switzerland; ^2^Department of Health Sciences and Technology, Institute for Biomechanics, Eidgenössische Technische Hochschule Zurich, Zurich, Switzerland; ^3^Division of Experimental Clinical Research, Vetsuisse Faculty, University of Bern, Bern, Switzerland; ^4^Tissue Engineering for Orthopaedics & Mechanobiology (TOM), Department for BioMedical Research (DBMR) of the Medical Faculty of the University of Bern, University of Bern, Bern, Switzerland; ^5^Department of Orthopedic Surgery and Traumatology, Inselspital Bern, University of Bern, Bern, Switzerland; ^6^Interfaculty Bioinformatics Unit, University of Bern, Bern, Switzerland; ^7^Swiss Institute of Bioinformatics, Lausanne, Switzerland; ^8^Research Office (Biostatistics), Paracelsus Medical University, Salzburg, Austria; ^9^Department of Ophthalmology and Optometry, Paracelsus Medical University Salzburg, Salzburg, Austria; ^10^Research Program Experimental Ophthalmology and Glaucoma Research, Paracelsus Medical University, Salzburg, Austria; ^11^Department of Biomedical Engineering, Rochester Institute of Technology, Rochester, NY, United States; ^12^Spine Center, Schön Clinic Munich Harlaching, Academic Teaching Hospital and Spine Research Institute of the Paracelus Medical University Salzburg, Munich, Germany

**Keywords:** IVDD, neuroinflammation, intervertebral disc, DAMPs, NP cells, fibronectin fragments

## Abstract

**Background:** Canine intervertebral disc disease (IVDD) represents a significant clinical problem in veterinary medicine, with similarities to the human pathology. Host-derived damage-associated molecular patterns like fibronectin fragments (FnF) that develop during tissue dysfunction may be of specific relevance to IVD pathologies by inducing an inflammatory response in resident cells.

**Aim:** This project aimed to determine the presence and pathobiological role of FnF during IVD herniation in dogs, with a focus on inflammation.

**Methods:** Herniated nucleus pulposus (NP) material from five dogs as well as non-herniated adjacent NP material from three dogs was collected during spinal surgery required due to acute IVD herniation. The presence of different types of FnF were determined by Western blot analysis. NP cells isolated from six herniated canine IVDs were then exposed to 30 kDa FnF. NP cell inflammation and catabolism was examined by investigating the expression of IL-1β, IL-6, IL-8, and COX-2, as well as MMP-1 and MMP-3 by qPCR (all targets) and ELISA (IL-6, PGE_2_).

**Results:** Amongst multiple sized FnF (30, 35, 45, and >170kDa), N-terminal fragments at a size of ~30 kDa were most consistently expressed in all five herniated IVDs. Importantly, these fragments were exclusively present in herniated, but not in non-herniated IVDs. Exposure of canine NP cells to 500 nM 30 kDa FnF caused a significant upregulation of IL-6 (62.5 ± 79.9, *p* = 0.032) and IL-8 (53.0 ± 75.7, *p* = 0.031) on the gene level, whereas IL-6 protein analysis was inconclusive. Donor-donor variation was observed in response to FnF treatment, whereby this phenomenon was most evident for COX-2, with three donors demonstrating a significant downregulation (0.67 ± 0.03, *p* = 0.003) and three donors showing upregulation (6.9 ± 5.5, *p* = 0.21). Co-treatment with Sparstolonin B, a TRL-2/TRL-4 antagonist, showed no statistical difference to FnF treatment alone in all tested target genes.

**Conclusion:** Given the presence of the 30 kDa FnF in canine herniated IVDs and the proinflammatory effect of 30 kDa FnF on NP cells, we concluded that the accumulation of FnF may be involved in the pathogenesis of canine IVDD. These results correspond to the findings in humans with IVDD.

## Introduction

Intervertebral disc (IVD) disease (IVDD), including painful degeneration and herniation, is considered a major cause of acute and chronic low back pain not only in humans but also in dogs (1, 2). Lower back pain is the main cause of disability in humans and therefore has a tremendous socio-economic impact. Similarly, in dogs, IVDD is a prevalent disorder and shares many similarities in the clinical presentation, but also potentially in its aetiopathogenesis (3, 4). While the molecular disease mechanisms in humans, specifically the mechanisms of inflammation during disc aging and degeneration (= inflammaging) are well-investigated (5–8), considerably less is known about the pathobiology of canine IVDD. Nevertheless, similar to the situation found with humans, we and others could demonstrate enhanced expression of proinflammatory cytokines in herniated canine IVDs compared to non-herniated IVD (9–11). The production and release of proinflammatory factors can be mediated through numerous stimuli, including pathogen recognition receptors such as toll-like receptors (TLRs), which are expressed on the surface of IVD cells (12). TLRs can be activated by pathogen-associated molecular patterns (PAMPs), such as lipopolysaccharide (LPS) and also by specific damage-associated molecular patterns (DAMPs) (13, 14). DAMPs are host-specific, cell- or tissue-derived biomolecules that are produced due to trauma, ischemia, and tissue damage. Unlike PAMPs, DAMPs do not require the presence of pathogens. The best known DAMP molecules include High-Mobility-Group-Protein B1 (HMGB1), heat shock proteins (HSPs), S100 and histones, but also certain matrix fragmentation products such as hyaluronic acid fragments or fibronectin fragments (15, 16). Fibronectin is a glycoprotein consisting of two subunits of ~220–250 kDa each, with high relevance in cell-to-cell and cell-to-matrix adhesion (17). Fibronectin undergoes proteolytic fragmentation upon exposure to numerous enzymes, resulting in different sized fragments with different biological functionality: N-terminal FnF (30 kDa, 70 kDa), Gelatin-binding 45 kDa FnF, Cell-binding 120 kDa FnF, C-terminal FnF (40 kDa) (18).

We and others have previously demonstrated that the expression of FnF is increased during IVD degeneration in humans (19, 20). Furthermore, FnF appear to induce degenerative and inflammatory processes characterized by an increased expression of proinflammatory molecules such as interleukin (IL)-6, IL-1β and increased activity of extracellular matrix degrading enzymes (21–23). These processes are likely mediated via TLRs, specifically TLR-2 and/or TLR-4, with downstream activation of nuclear factor kappa-light-chain-enhancer of activated B-cells (NF-κB) and mitogen-activated protein (MAPK). Therefore, FnF may be important modulators of degeneration and inflammaging (via disc cell inflammation) during canine disc disease.

The aim of this project was to identify the presence of FnF in IVD material from dogs suffering from IVD herniation. Furthermore, we aimed to determine the inflammatory and catabolic response of canine IVD cells upon exposure to 30 kDa FnF, with and without inhibition of TLR-2/TLR-4, to gain mechanistic insights in the pathobiology of IVDD.

## Materials and Methods

### Expression of FnF in Herniated and Non-herniated Intervertebral Discs

#### Sample Collection

NP material was collected from client-owned dogs undergoing routine spinal surgery at the Small Animal Clinic of the Vetsuisse Faculty of the University of Bern, Switzerland due to acute onset of an intervertebral disc herniation (Table 1). A board-certified neurologist examined all dogs. After the lesion was localized, patients were stabilized with individual pain management therapy and further diagnostic workup was conducted. The patients were included in the study if the diagnosis of an acute IVD herniation was confirmed by an MRI. Patients presenting any signs of an illness other than IVDD were excluded from the study. Anesthesia was performed following a standard protocol, including intramuscular sedation with methadone (0.2 mg/kg) and acepromazine (0.03 mg/kg, Arovet AG, 8953 Dietikon, Switzerland), induction with intravenous propofol (2–8 mg/kg to effect, 16NC3652, Fresenius Kabi, 6370 Oberdorf, Switzerland) and maintenance with isoflurane in an air-oxygen combination, delivered with a rebreathing system (1.2–2% end-tidal isoflurane concentration). Subsequent routine ventral slot (cervical disc herniation) or hemilaminectomy (thoracolumbar disc herniation) was performed and herniated NP material retrieved from the spinal canal. Prophylactic fenestration (only in the thoracolumbar spine, but not in the cervical spine) of the adjacent IVD was performed as it might be prone to a future herniation (24–26). While NP material collected from the spinal canal formed the diseased IVD group, NP received from adjacent IVD served as non-herniated controls. The non-herniated control tissue was obtained from the same dogs that underwent surgery for the herniated disc, but from an adjacent disc via fenestration. The degree of IVD degeneration was assessed by high-field MRI images using the Pfirrmann grading system (27, 28). Collected herniated NP and non-herniated (control) NP material was shock frozen in liquid nitrogen and stored at−80°C for analysis of fibronectin fragmentation. For cell isolation, NP material was collected in DMEM/F12 (31,330-038, Thermo Scientific, Waltham, MA, USA) with 3% Antibiotic-Antimycotic (15,240,062, Gibco Life Technologies, Switzerland). More details about the cell isolation are provided in section Effects of Human 30 kDa FnF on Canine IVD Cells *in vitro* below). Informed written owner consent was obtained for each dog. Every procedure performed for this study was approved by the commission of animal experimentation of the canton of Bern, Switzerland (31496).

**Table 1:** Signalement and clinical presentation of the dogs included into the study.

**Nb**	**Breed**	**Gender**	**Onset (days)**	**Clinical presentation**	**Age at surgery/blood donation**
	**Candidates for western blot**				
**Affected**					
1	French bulldog	Female intact	1	Tetraplegia	6 years,8 months,5 days
2	Dachshund	Male intact	1	Non-ambulatory paraparesis	7 years,11 months,28 days
3	French bulldog	Male castrated	0.5	Non-ambulatory paraparesis	5 years,8 months,17 days
4	Toy poodle	Male castrated	3	Paraplegia without deep pain	11 years,5 months,0 days
5	French bulldog	Male intact	3	Ambulatory paraparesis	3 years,10 months,16 days
**Controls**					
4	Toy poodle	Male castrated	3	Paraplegia without deep pain	11 years,5 months,0 days
6	Dachshund	Female spayed	6	Non-ambulatory paraparesis	6 years,8 months,2 days
7	American staffordshire terrier	Male castrated	3	Non-ambulatory paraparesis	7 years,10 months,14 days
	**Candidates for IVD cell cultures**				
8	French bulldog	Male intact	1	Ambulatory paraparesis	8 years, 2 days
9	French bulldog	Male castrated	1	Ambulatory paraparesis	6 years, 9 months, 29 days
10^*^	French bulldog	Male intact	1	Pain	6 years, 4 days
11^*^	French bulldog	Male intact	1	Pain	6 years, 4 days
12	Beagle	Female intact	1	Non-ambulatory tetraparesis	8 years, 8 months, 6 days
13	Maltese	Female spayed	1	Ambulatory paraparesis	3 years, 8 months, 27 days

*Summary of the total study population. NP material collected from the spinal canal formed the diseased IVD group, NP collected from adjacent IVD served as healthy controls*.

* *This dog had two simultaneous herniations at two different sites*.

#### Protein Extraction

For protein isolation, frozen NP specimens were pulverized using a custom-made cryo-pulverizer (mortar and pestle), then placed into RIPA buffer (89,900, Thermo Scientific), 0.5 M EDTA Solution 10 × (1,860,851, Thermo Scientific) and Halt Protease Inhibitor Single-Use Cocktail (78,430, Thermo Scientific) and incubated on ice for 30 min on an orbital shaker. Samples then were further ground using a Polytron PT 2500E (Kinematica AG, 6014 Lucerne, Switzerland) before final centrifugation at 12,000 × g for 20 min at 4°C. The supernatant of the samples was transferred to a new tube, and the total protein content was quantified by standard Bradford's Protein Assay (5,000,006, BIO-RAD Laboratories Inc., California 94547, USA) according to the manufacturer's recommendations. Total protein concentrations of the samples were calculated using the values from the standards as references.

#### Western Blotting

Twenty micrograms of each protein sample was boiled in 4 × Laemmli Sample Buffer (1,610,747, BIO-RAD) without β-mercaptoethanol at 95°C for 2 min on an orbital shaker at 300 rpm. As a positive control, the 30 kDa proteolytic fragments from human plasma fibronectin (F9911, Sigma-Aldrich Chemie GmbH, 9471 Buchs SG, Switzerland) were used in a concentration of 1 × 10^−4^ μg and treated equally. 4–15% Mini-PROTEAN® TGX Stain-Free™ Protein Gels, ten well, 30 μL (4,568,083, BIO-RAD) were loaded with 1.5 μl page ruler^TM^ Pre-stained protein ladder (26,616, Thermo Scientific), 1 × 10^−4^ μg of the positive control and 20 μg of each sample. Proteins were separated by electrophoresis at 130 V for 80 min and then electroblotted onto Trans-Blot® Turbo™ Mini PVDF transfer packs (1,704,156, BIO-RAD) using the Trans-Blot® Turbo^TM^ Transfer-System (BIO-RAD). Appropriate loading was verified by imaging the membranes with the Chemidoc^TM^ Touch Imaging System (BIO-RAD) prior to blocking with 3% bovine serum albumin (BSA, A7906, Sigma-Aldrich) in tris-buffered saline-tween (TBS-T) at room temperature for 1 h. Before incubation with the primary antibody, the membranes were washed three times with TBS-T for 10 min. The primary monoclonal mouse anti-fibronectin antibody-specific to the N-terminus of fibronectin (7D5, provided by Prof. Deane Mosher, University of Wisconsin), was applied in a concentration of 1:1,000 in 3% BSA, in TBS-T at 4°C, overnight. Before incubation with the secondary anti-mouse horseradish peroxidase linked antibody (7076, Cell Signaling Technology®, 2,316 WZ Leiden, Netherlands) in a concentration of 1: 10,000 in 3% BSA/TBS-T at room temperature for 1 h, the membranes were washed three times with TBS-T. After three final 10 min washing steps in TBS-T, chemiluminescence was detected using SuperSignal^TM^ West Pico PLUS Chemiluminescent Substrate (34,577, Thermo Scientific). Images were processed and analyzed using Chemidoc^TM^ Touch Imaging System (BIO-RAD).

### Effects of Human 30 kDa FnF on Canine IVD Cells *in vitro*

#### Isolation of IVD Cells

Canine IVD cells were isolated from six patients (Table 1) undergoing spinal surgery (hemilaminectomy) due to an acute thoracolumbar IVD herniation as described above. Harvested IVD tissue was immediately immersed in serum-free transport medium (DMEM/F12), supplemented with 3% Antibiotic-Antimycotic. The harvested IVD tissue was then enzymatically digested using 0.2% collagenase NB4 (S1745401, Nordmark Biochemicals, 25436 Uetersen, Germany) and 0.3% dispase II (13437500, Sigma-Aldrich) dissolved in phosphate buffered saline (PBS, 09-8912-100, Medicago AB, SE-755 98 Uppsala, Sweden) for 6 h at 37°C. After the enzymatic isolation, the cells were expanded in growth medium consisting of DMEM/F12, supplemented with 10% fetal calf serum (FCS, F7524, Sigma-Aldrich) and 1% Antibiotic-Antimycotic at 37°C with 5% CO_2_ up to the fourth passage.

#### Exposure of Canine IVD Cells to 30 kDa Fibronectin Fragments

For the stimulation experiments, IVD cells were cultured for 24 h in a 6-well plate at a density of 2 × 10^5^ cells/well in DMEM/F12 supplemented with 1% Antibiotic-Antimycotic and 10% FCS. On the next day, cells were serum starved in media supplemented with Polymyxin B (100 units/mL, 81271, Sigma-Aldrich) with or without Sparstolonin B (SsnB, 25 μM, TRL-2/TRL-4 inhibitor, SML1767, Sigma-Aldrich) for 2 h prior to stimulation with FnF. Afterwards, cells were treated with 30 kDa proteolytic fragments from human plasma fibronectin (FnF, 500 nmol/L, F9911, Sigma-Aldrich) or recombinant canine IL-1β, which was used as positive inflammation control (10 ng/ml, 3747-CL-025, R&D Systems, Inc., Minneapolis, MN 55413, USA) for 18h. Untreated NP cells served as a negative control group, NP cells treated with Sparstolonin B alone were used as a control to the combination treatment of FnF, and Sparstolonin B. Used concentrations were based on the range of FnF found in IVDs during degeneration (19, 29). After the incubation time, media supernatants were collected, and cells were lysed using RLT Lysis Buffer (79,216, Qiagen, Hilden, Germany). Both cell lysates and supernatants were stored at −80°C until further analysis.

#### Reverse Transcription

Total RNA from IVD cell lysates was isolated and purified using the RNeasy Mini Kit (74,104, Qiagen) according to the manufacturer's protocol without adjustments. Quantity and quality of RNA were assessed using the NanoDrop™ 1000 Spectrophotometer (Thermo Scientific). One μg total RNA was reverse transcribed to cDNA using the reverse transcription reagents from TaqMan® (N808-0234, Applied Biosystems, Foster City, CA, USA). Briefly, one sample consisted of 6.6 μL of 10× Taqman buffer (4,311,235, Applied Biosystems), 14.52 μL of 25 mM MgCl_2_ (100,020,476, Applied Biosystems), 13.2 μL dNTP mix (45–100, Applied Biosystems), 3.3 μL of Random hexamer (N8080127, Thermo Scientific), 1.32 μL of RNase inhibitor (100,021,540, Applied Biosystems), 1.65 μL of reverse transcriptase (4,311,235, Applied Biosystems) that was added to 1 μg of total RNA. Finally, the volume was adjusted to 60 μL with RNase free water (1018017, Qiagen) and reverse-transcribed by the T100^TM^ Thermal Cycler (BIO-RAD) performing 10 min cycle at 25 °C, followed by 120 min cycle at 37°C. cDNA samples were then stored at −20°C until further analysis.

#### Quantitative RT-PCR (qPCR)

For qPCR, each reaction mix consisted of cDNA (10 ng/well) diluted with RNase free Water (1,018,017, Qiagen) to a total volume of 4.5 μL, TaqMan® Fast Universal PCR Master Mix 2× (5 μL/well, 4367846, Applied Biosystems) and canine-specific TaqMan primers (0.5 μL/well, Table 2). Real-time analysis of mRNA expression was quantified using C1000^TM^ Touch Thermal Cycler, CFX96^TM^ Real-Time System (BIO-RAD). Each reaction was performed in duplicates in a Micro Amp® Fast Optical 96-Well Reaction Plate 0.1 mL (4,346,906, Applied Biosystems) with the amplification condition set to one cycle at 95°C for 20 s, followed by 44 cycles of 95°C for one second and 60°C for 20 s. Data processing of the obtained C*q*-values was conducted using the 2^−ΔΔCT^ method and presented as the fold change in gene expression normalized to the reference gene GAPDH and relative to a control condition (30). Cultures treated with fibronectin fragments were normalized to the untreated controls, and samples treated with fibronectin fragments and Sparstolonin B were normalized to samples with Sparstolonin B alone.

**Table 2:** Identification numbers of TaqMan primers (TaqMan® Gene Expression Assays, Thermo Scientific) used in the present study.

**Target gene**	**Assay ID**	**Correlation to inflammatory process**
GAPDH	Cf04419463_gH	Reference gene
IL-1β	Cf02671951_g1	Proinflammatory cytokine (5)
IL-6	Cf02624153_m1	Proinflammatory cytokine (5)
IL-8	Cf02624283_m1	Proinflammatory cytokine (5)
MMP1	Cf02651000_g1	Collagen (I, II, III) cleavage (57)
MMP3	Cf02625960_m1	Proteoglycan and collagen (II, III) cleavage (57)
COX-2	Cf02680566_gH	Mediator of pain (58)

*GAPDH, Glyceraldehyde 3-phosphate dehydrogenase; IL-1β, interleukin 1 beta; IL-6, interleukin 6; IL-8, interleukin 8; MMP1, matrix metalloproteinase 1; MMP3, matrix metalloproteinase 3; COX-2, cyclooxygenase-2*.

#### Enzyme-Linked Immunosorbent Assay (ELISA)

Secreted IL-6 and PGE_2_ were evaluated in the cell culture supernatant by commercially available ELISA kits (CA6000, R&D Systems, Minneapolis, MN. USA; KGE004B, R&D Systems) following the manufacturer's protocol. Samples were used undiluted, and each sample was tested in duplicates. The absorbance was measured (Tecan, Infinite M200 PRO) at 450 nm (with subtracted 540 nm absorbance). IL-6 and PGE_2_ concentrations were calculated based on the standard curve. Results are presented as pg/mL.

#### Statistical Analysis

Data were checked for consistency and normality. Paired one and two sample tests based on classical statistics (*t*-tests), bootstrap methods (bootstrap *t*-tests) as well as non-parametric tests (Wilcoxon signed-rank test) were used to analyse the data. A significance level of 5% was taken to be statistically significant. Whisker plots were used to illustrate the results. All statistical analyses in this report were performed by the use of NCSS (NCSS 10, NCSS, LLC. Kaysville, UT) and STATISTICA 13 (Hill, T. & Lewicki, P. Statistics: Methods and Applications. StatSoft, Tulsa, OK).

## Results

### Study Population

A total number of 13 client-owned dogs were enrolled in the present study (Table 1).

Seven dogs with an acute IVD herniation were investigated for the presence of FnF. Five herniated IVDs served as the diseased samples, and three non-herniated IVDs served as controls. The study population consisted mainly of chondrodystrophic small breed dogs, which are most often affected by IVDD. Due to the small size of these canine patients, the surgically obtained sample volumes were minimal. Therefore, sufficient material from both the herniated (hemilaminectomy; diseased), but also a non-herniated IVD (fenestration; control), could only be collected from one of the seven dogs. In four dogs, the quantity of the material collected from the herniated IVD, but not from the non-herniated site, was sufficient for the experiments. In two dogs, it was possible to obtain enough material from a non-herniated, but not from the herniated IVD. The Pfirrmann grade of the IVDs in the control group (non-herniated IVD material collected by fenestration) was consistently lower (grade III) than the IVD in the diseased group (grade V).

For the NP cell isolation and culture, herniated IVD material from another 6 dogs was used and investigated for their inflammatory response upon exposure to FnF.

### Expression of Fibronectin Fragments in Herniated Intervertebral Disc Material

**Figure 1:**
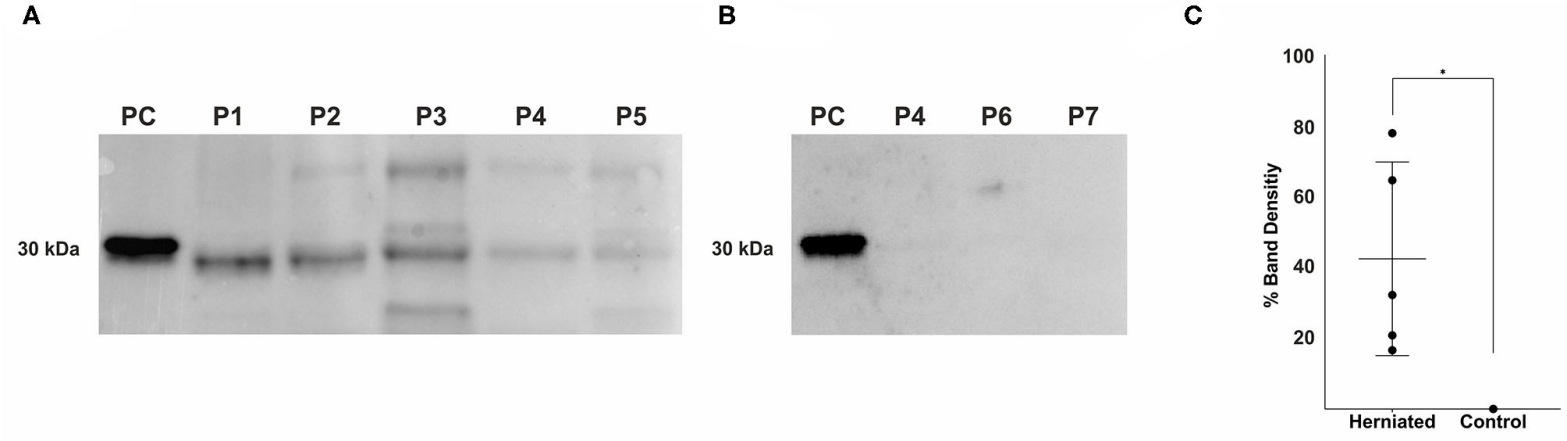
Detection of fibronectin fragments in herniated and non-herniated canine nucleus pulposus material. FnF were detected using a mouse monoclonal antibody specific for the N-terminal end of fibronectin. **(A)** Lane 1: 1 × 10^−4^ μg, 30 kDa proteolytic fragments from human plasma fibronectin (F9911, Sigma-Aldrich); Lane 2: Patient 1; Lane 3: Patient 2; Lane 4: Patient 3; Lane 5: Patient 4; Lane 6, Patient 5; **(B)** Lane 1: 1 × 10^−4^ μg, 30 kDa proteolytic fragments from human plasma fibronectin (F9911, Sigma-Aldrich); Lane 2: Patient 4; Lane 3: Patient 6; Lane 4: Patient 7; (for patient description see Table 1); **(C)** Density values of the bands between 27 and 29.2 kDa of all donors. Black points represent the individual bands of the corresponding donors **(A,B)** and their respective signal intensity in relation to the positive control group (Band 1: 30 kDa FnF). Data is presented as mean and 95% CI. *denotes statistically significant differences with *p* < 0.05.

Western blot analysis of herniated IVD material with the mouse anti-fibronectin antibody specific to the N-terminus of fibronectin (antibody 7D5, kindly provided by Prof. Deane Mosher) revealed multiple sized FnF (Figure 1). All herniated IVD specimens showed one band at a molecular weight at ~30 kDa. Moreover, three herniated IVDs (patients 3, 4, and 5) also displayed bands at a molecular weight of around 44 kDa, and one herniated NP (patient 3) showed a band at a molecular weight of ~35 kDa. Additionally, 170 kDa fragments were infrequently detected in herniated IVDs. Importantly, no FnF were detected in the three non-herniated control IVD samples (Figure 1).

### Effects of 30 kDa FnF Stimulation (Gene Expression)

**Figure 2:**
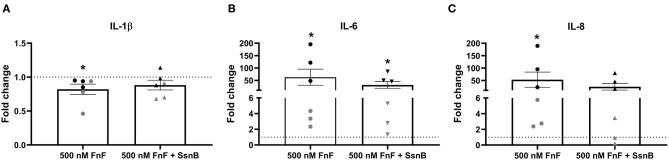
Gene expression of IL-1β **(A)**, IL-6 **(B)**, and IL-8 **(C)** after 18 h exposure to 30 kDa fibronectin fragments (500 nM) alone and in combination with the TRL-2/TRL-4 inhibitor Sparstolonin B. Values were normalized to the reference gene (GAPDH) and are shown as fold change compared to untreated control (for 500 nM FnF) or Sparstolonin B alone (for 500 nm FnF + SsnB). *Denotes statistically significant differences with *p* < 0.05. Black symbols indicate high responders (*n* = 3) and gray symbols indicate low responders (*n* = 3) (*n* = 6 total). Points demonstrate NP cells treated with 500 nM 30 kDa FnF. Triangles demonstrate NP cells treated with a combination of FnF and Sparstolonin B.

**Figure 3:**
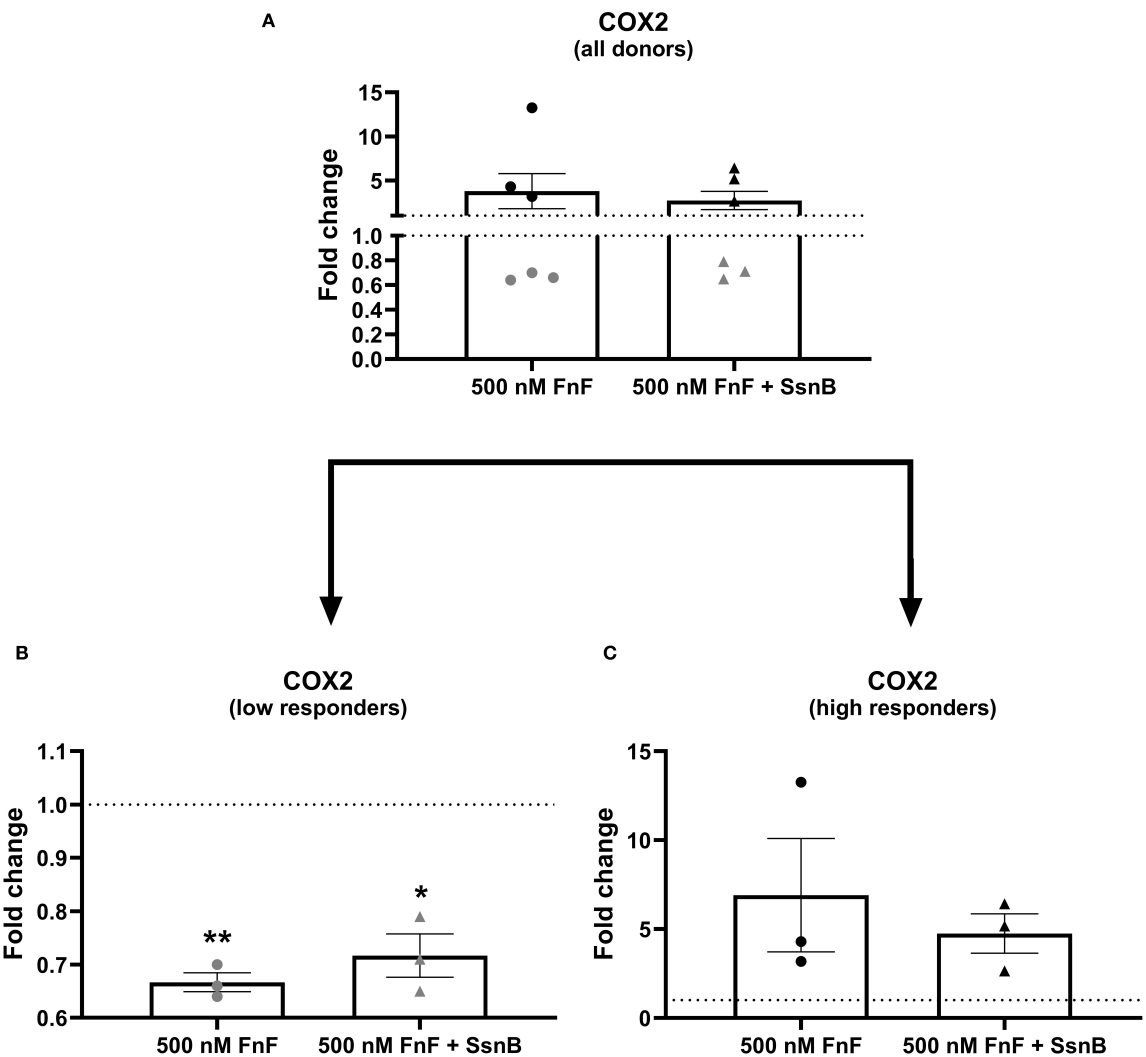
Gene expression of COX-2 after 18 h exposure to 30 kDa fibronectin fragments (500 nM) alone and in combination with the TRL-2/TRL-4 inhibitor Sparstolonin B. All donors **(A)** and split according to low responders **(B)** and high responders **(C)**. mRNA was quantified by real time RT-qPCR (RT-qPCR). Values of COX-2 were normalized to the reference gene (GAPDH) and presented as the fold change compared to untreated control (FnF stimulation alone) or NP cells treated with Sparstolonin B alone. *Denotes statistically significant differences with *p* < 0.05. Due to significant donor variation, low and high responders are displayed in a separate graph **(B,C)**. Points demonstrate NP cells treated with 500 nM 30 kDa FnF. Triangles demonstrate NP cells treated with a combination of FnF and Sparstolonin B. The color black indicates high responders and the color gray indicates low responders. *Denotes statistically significant differences with *p* < 0.05 and **p < 0.01.

**Figure 4:**
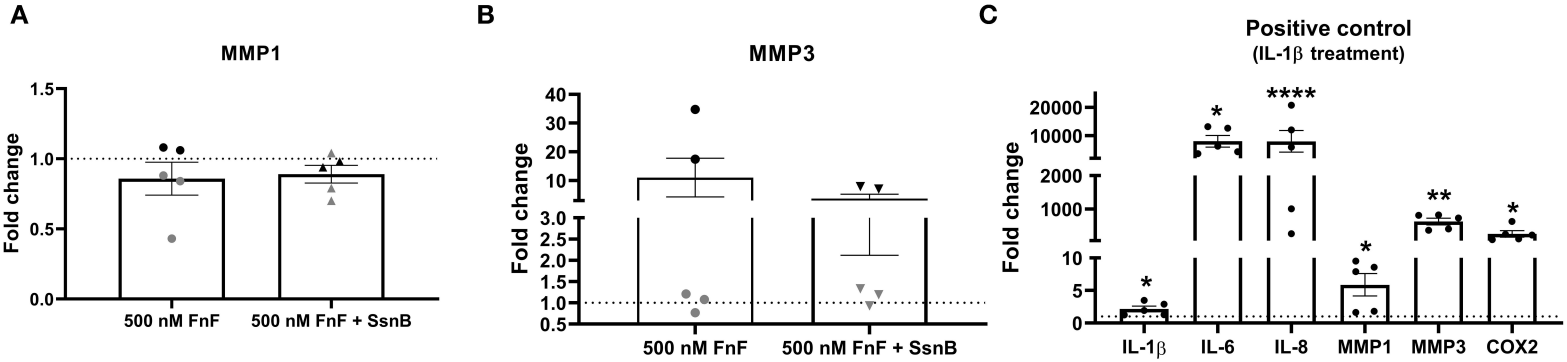
Gene expression of MMP-1 **(A)** and MMP-3 **(B)** after 18 h exposure to 30 kDa fibronectin fragments (500nM) alone and in combination with the TRL-2/TRL-4 inhibitor Sparstolonin B. Gene expression of IL-1β, IL-6, IL-8, COX-2, MMP-1, and MMP-3 after 8h exposure to canine IL-1β **(C)**. mRNA was quantified by real time RT-qPCR (RT-qPCR). Values of MMP-1 and MMP-3 were normalized to the reference gene (GAPDH) and presented as the fold change compared to untreated control (FnF stimulation alone) or NP cells treated with Sparstolonin B alone **(A,B)**. Values of IL-1β, IL-6, IL-8, MMP-1, MMP-3, and COX-2 were normalized to the reference gene (GAPDH) and presented as the fold change compared between IL-1β treatment and untreated controls **(C)**. *Denotes statistically significant differences with *p* < 0.05. Points demonstrate NP-cells treated with 500nM 30kDa FnF. Triangles demonstrate NP cells treated with a combination of FnF and Sparstolonin B. The color black indicates high responders and the color gray indicates low responders. *Denotes statistically significant differences with *p* < 0.05, **p < 0.01 and ****p < 0.0001.

To study the effect of FnF on IVD inflammation, NP cells were stimulated with 500 nM 30 kDa fragments of plasma fibronectin (alone or in combination with the TRL-2/TRL-4 inhibitor Sparstolonin B) for 18 h. Gene expressions changes are presented in Figure 2 (IL-1β, IL-6, IL-8), Figure 3 (COX-2), and Figure 4 (MMP-1, MMP-3, positive control).

FnF treatment led to a statistically significant upregulation of IL-6 (*p* = 0.032, Figure 2B) and IL-8 (*p* = 0.031, Figure 2C) as compared to the untreated control. Donor-donor variation was evident in response to FnF treatment and is indicated with black and gray colors, with three donors showing a small to moderate response (3.3 ± 0.1 for IL-6; 3.6 ± 1.8 for IL-8), and three donors showing a moderate to strong response (121.7 ± 73.8 for IL-6; 102.3 ± 83.8 for IL-8, respectively). IL-1β mRNA demonstrated negligible downregulation (*p* = 0.036, Figure 2A). The responder phenomenon was most evident for COX-2 mRNA expression, where three out of six donors demonstrated a statistically significant downregulation (0.67 ± 0.03, *p* = 0.003, Figure 3B). In contrast, the other three donors showed a mild trend toward an upregulation (not statistically significant, 6.9 ± 5.5, *p* = 0.21, Figure 3C). FnF treatment did however not result in a significant change in MMP-1 (*p* = 0.29, Figure 4A) or MMP-3 (*p* = 0.21, Figure 4B) mRNA expression.

Co-treatment with Sparstolonin B (TRL-2/TRL-4 antagonist) did not show any statistical difference compared to FnF treatment alone in all tested gene targets (Figures 2–4).

When NP cells were stimulated with IL-1β as a proinflammatory control, they exerted a strong and statistically significant upregulation of all measured markers, as shown in Figure 4C (IL-1β *p* = 0.026; IL-6 *p* = 0.011; IL-8 *p* = 0.0001; MMP-1 *p* = 0.047; MMP-3 *p* = 0.003; COX-2 *p* = 0.022).

### Effects of 30 kDa FnF Stimulation (Protein Expression)

IL-6 release into the cell culture medium was detected in the positive control (IL-1β treatment) in all donors (Supplementary Figure 3A). However, it could only be detected in two out of six donors in the 500 nM FnF group (2.19 and 2.76 pg/mL) and the 500 nM FnF group + 25 μM SSnB (5.84 and 14.8 pg/mL), as well as in one out of six donors in the 25 μM SSnB group (0.57 pg/mL).

Due to the limited range of the sensitivity (39.0–2,500 pg/mL) of the PGE_2_ ELISA kit, the release of PGE_2_ (as a product of COX-2) could not be detected in any of the tested samples except for the positive control (IL-1β treatment, Supplementary Figure 3B).

## Discussion

It is well-established that canine IVD herniation is the result of a course of complex biomechanical and biological interactions that resemble the human pathology (31–34). However, the current understanding of the exact molecular patho-mechanisms of IVD degeneration and herniation in dogs is still poor. To the best of our knowledge, no study has thus far been conducted on the role of DAMPs in canine IVDD. In human patients, extracellular matrix (ECM) degradation through proteases and inflammation, which can (amongst other mechanisms) be induced by degeneration-associated DAMPs (12, 35, 36), contribute to degenerative disc disease.

Amongst the various ECM fragments, FnF have attracted much attention over the past decades as research in rodents and humans has provided clear evidence for the presence of FnF during IVD degeneration (22, 23, 37). Importantly, FnF potentially contribute to the disease through induction of matrix degradation and inflammation and suppression of proteoglycan synthesis, specifically through the 30 kDa N-terminal FnF (22, 23, 37, 38). In the present study on canine IVDs, we were not only able to demonstrate multiple sized FnF in herniated canine NP material, but also their potential proinflammatory activity on a cellular level. Within the retrieved specimens of herniated canine IVDs, FnF with a molecular weight of around 30 kDa were most consistently present. Importantly, we could not detect any FnF within NP material that was not herniated, as shown in Figure 1. The observed molecular weight variance of the bands around 30 kDa between samples and the positive control (30 kDa proteolytic fragments from human plasma fibronectin) could therefore be associated with donor dependent progressive protein degradation or alternative splice variants during the degeneration of the extracellular matrix of the IVD (39, 40). We also found FnF with a molecular weight of ~45 and 35 kDa in herniated canine IVDs. Stanton et al. (41) showed that a 45 kDa collagen-binding fragment of Fn can induce MMP-13 synthesis in porcine chondrocytes and therefore demonstrates catabolic properties. FnF of 35 kDa size have thus far not been specifically described in the context of tissue dysfunction or degeneration but may represent an intermediate fragmentation product, similar to the larger fragments (>170 kDa) that were infrequently present.

The 30 kDa FnF, which was the most abundant size in the current study, has previously been shown to have degradative and inflammatory properties during human IVDD *in vitro* (20) and during rabbit IVDD *in vivo* (37). To demonstrate not only the presence but also the proinflammatory and degradative capacity of 30 kDa FnF, IL-1β, IL-6, IL-8, and COX-2 as well as MMP-1, MMP-3–cytokines and matrix degrading enzymes known to promote matrix degradation and chemokine production during IVDD–were measured in canine NP cells upon FnF treatment (42). We are able to show that 30kDa FnF significantly increased gene expression of IL-6, IL-8, and partially COX-2 compared to control NP cells, whereas minor or non-significant changes were observed for IL-1β, MMP-1 and MMP-3.

The induction of IL-6 and IL-8 in canine NP cells is similar to previously described results in human IVD cells (21). The involvement of IL-6 and IL-8 in the pathobiology of IVD degeneration is well-established. One study in dogs showed an upregulation in the gene expression of IL-8 and downregulation in the gene expression of IL-6 during IVDD (11). Increased levels of IL-6 and IL-8 in IVD in humans correlate with degeneration and inflammation within the IVD (36, 43–46). Aside from its catabolic effects on NP cells, IL-6 is also involved in the development of pain during the course of IVDD (47). IL-8 is the classical activator of neutrophils and has also been detected in increased levels during IVDD in humans (45).

Interestingly, we observed that three donors demonstrated a low to moderate response to 30 kDa FnF, whereas three donors responded in a clear manner. The three donors with the less pronounced response pattern were cryopreserved cells, indicating that the process of cryopreservation may affect their sensitivity to FnF. It is known; from work with germ cells that cryopreservation can lead to genomic alterations or damage, for example, deletions, base modifications or crosslinks on the DNA, which, therefore, could explain an altered reaction pattern between fresh and cryopreserved cells (48, 49). A study evaluating the effect of cryopreservation on the viability of canine and human NP cells did not show differences in proliferation capacity, cell viability, and ability to produce extracellular matrix. However, this study did not measure the potential of the NP cells to react to proinflammatory stimuli (50).

COX-2 is a key enzyme in the regulation of PGE_2_ synthesis and plays an important role in inflammation. It is induced in many types of cells by the stimulation of inflammatory cytokines such as IL-1β and tumor necrosis factor alpha (TNF-α) and has been confirmed in IVD material from humans with lumbar IVD herniation (51). In this study, COX-2 showed disparate mRNA response patterns between fresh and frozen cells, with FnF significantly inhibiting or tendentially inducing COX-2 expression, respectively.

As the effect of 30kDa FnF exposure on the gene expression of canine NP cells was most promising for IL-6 and COX-2, these markers were also tested on the protein level. However, results were inconclusive due to detectability issues in untreated controls, and FnF stimulated cells. However, exposure of NP cells to the positive control IL-1β led to a marked increase in the production of both measured markers, illustrating that the assays functioned appropriately. The use of more sensitive tests or the analysis of protein levels at later treatment time points might help to circumvent the observed detection problems.

After the confirmation of the inflammatory potential of 30 kDa FnF, we aimed to identify the responsible underlying molecular mechanism. The relevance of the TLR pathway in IVD degeneration has been demonstrated in previous investigations (52, 53). Although FnF have been described as modulates of TLR-2 and TLR-4 in various cell types, for example NP cells (35), articular chondrocytes (54), and mast cells (55), we did not observe any reduction of FnF induced inflammation with SSnB co-treatment. SSnB was shown to selectively block TLR-2 and TLR-4 by inhibiting the early intracellular steps in TLR-2 and TLR-4 signaling. It does not block TLR-3 and TLR-9 (56). Interestingly, previous work on human IVD disc inflammation with *Cutibacterium acnes* reported involvement of TRL-2/TRL-4 in IVD inflammation, but only in a subset of patients (57). Besides that, it could be possible that the 30 kDa FnF, which is specific for TRL-2/TRL-4 in humans (35), signals through other receptors in dogs, such as the NOD2 (nucleotide-binding and oligomerization domain) pathways, as shown in human articular chondrocytes (58).

In this study, we compared herniated and non-herniated NP tissue, with the non-herniated NP materials showing a lower degeneration grade throughout. Ideally, the non-herniated control IVD material would have consisted of healthy, non-degenerated IVDs. In the clinical setting of client-owned dogs; however, this is an unrealistic requirement as the majority of dogs undergoing surgery for IVD herniation are chondrodystrophic and therefore suffer from various stages of generalized IVDD. A different study set-up using control dogs with healthy IVD would be necessary to investigate the situation in non-degenerated IVD reliably. In a human study, significantly elevated levels of fibronectin were present only in higher grades of IVD degeneration (19). This is, therefore, in concordance with the findings in our study, as FnF were detected only in the diseased, herniated IVD but not in the non-herniated control IVD, despite the presence of lower grade signs of degeneration in this group. The herniation itself is a traumatic event, which often involves disruption of local spinal blood vessels with subsequent hemorrhage. Fibronectin is not only a protein abundantly present in the ECM, but is also a blood component. Although gross macroscopic evaluation did not show the presence of blood in the collected tissue samples, we cannot exclude the presence of blood. However, as FnF were also detected in human degenerated IVDs (19) without herniation, i.e., in poorly vascularized structures (59), we believe that the detected FnF originate from the herniated IVD tissue.

It is important to note that we used commercially available human 30 kDa FnF for the cell stimulation experiments. Performing human and canine fibronectin amino acid alignment of the Fn1 subunit of fibronectin with Basic Local Alignment Search Tool (BLAST, https://blast.ncbi.nlm.nih.gov/Blast.cgi) shows ~70.6% identity. In contrast, if comparing only the 30 kDa N-terminal end of human and canine fibronectin, an amino acid alignment of ~99.55% was found. The suitability of using human 30 kDa FnF to treat canine NP cells is evidenced by the observed response patterns, specifically the significant induction of IL-6 and IL-8. The concentration of FnF used in our study was based on concentrations used in studies of human IVDD (29). Ruel et al. (29) estimated the naturally occurring concentrations of FnF in degenerated human IVDs at nanomolar levels, by the signal intensity during western blot. Aside from NP cells, invading macrophages during IVD herniation may also be sensitive to FnF. Certain FnF can affect macrophage activation and polarization (60) and compared to NP cells. Macrophages are more potent immune modulators. Subjected to an adequate stimulus, macrophages are known to be able to produce a variety of proinflammatory cytokines, including TNFα, IL-1β, and IL-6. They are thought to be the dominant regulator of the secretion of MMP-1 and MMP-3 (61–64). Recently we could demonstrate the occurrence of both M1 inflammatory macrophages and M2 pro-healing macrophages in herniated IVDs, but not in healthy control IVDs of dogs (unpublished data) (65). Therefore, further work on the reaction of macrophages upon exposure to FnF might provide valuable insights into the pathobiology of canine IVDD.

## Conclusion

In conclusion, we were able to demonstrate the presence of FnF of various sizes in canine IVDs suffering from acute IVD herniation, specifically those of ~30 kDa. Furthermore, exposure of NP cells to 30 kDa FnF resulted in increased gene expression of proinflammatory cytokines (IL-6 and IL-8). This finding indicates that the accumulation of FnF in the canine IVD might be involved in maintaining and promoting IVD degeneration and inflammation. Future work must be implemented to assess the impact of FnF accumulation in the pathobiology of IVDD. Furthermore, the involvement of immune cells, especially macrophages, in FnF induced inflammation, should be included in future studies.

## Data Availability Statement

All data generated and analyzed during this study are included in this published article or the supplementary material.

## Ethics Statement

The animal study was reviewed and approved by Commission of Animal Experimentation, Veterinärdienst of the Canton of Bern, Herrengasse 1, 3011 Bern, Switzerland. Written informed consent was obtained from the owners for the participation of their animals in this study.

## Author Contributions

MS collected the samples, conducted the laboratory work, analyzed and merged the data, reviewed the literature, and drafted the manuscript. AS performed NP cell culture experiments and helped to draft the manuscript. IC, BG, and HL helped to design the study and draft the manuscript. BG also acquired funding. SF helped design the study, supervised the experiments, and reviewed the manuscript. WH performed the statistical analysis of the cell culture experiments. FF acquired funding, developed the study design, helped with the organization of the laboratory work, reviewed the final paper, and operating surgeon collecting tissue specimens during hemilaminectomy surgery. KW-K acquired funding, designed the study, helped with and supervised the execution of laboratory work, as well as the statistical interpretation of the data, and reviewed the final paper. All authors reviewed and approved the final manuscript.

## Conflict of Interest

The authors declare that the research was conducted in the absence of any commercial or financial relationships that could be construed as a potential conflict of interest.

## Abbreviations

BLAST: basic local alignment search tool
BSA: bovine serum albumin
COX: cyclooxygenase
DAMPS: damage-associated molecular patterns
DMEM: dulbecco's modified eagle's medium
ECM: extracellular matrix
EDTA: ethylenediaminetetraacetic
ELISA: enzyme-linked immunosorbent assay
Fn: fibronectin
FnF: fibronectin fragments
HSPs: heat shock proteins
IL: interleukin
INF: interferon
IVD: intervertebral disc
IVDD: intervertebral disc disease
kDa: kilodalton
LPS: lipopolysaccharide
MAPK: mitogen-activated protein kinase
MMP: matrix metalloproteinases
MRI: magnetic resonance imaging
mRNA: messenger ribonucleic acid
NF-kβ: nuclear factor kappa-light-chain-enhancer of activated B cells
NP: nucleus pulposus
PAMPS: pathogen-associated molecular patterns
PBS: phosphate buffered saline
PGE_2_: prostagalandine E_2_
PVDF: polyvinylidenfluorid
RNA: ribonucleic acid
RT-qPCR: quantitative real-time reverse transcriptase polymerase chain reaction
TBS-T: tris-buffered saline-tween
TLR: toll-like-receptor
TNF: tumor necrosis factor.
