# Supplementary material for: Fibronectin Fragments and Inflammation During Canine Intervertebral Disc Disease
Source: Front Vet Sci. 2020 Nov 16;7:547644. doi: 10.3389/fvets.2020.547644 (PMC7701143; doi:10.3389/fvets.2020.547644)
Supplement: Supplementary file 1 [file Data_Sheet_1.docx]

Supplementary Material

# Supplementary Figure 1: Stain free blot of herniated and non-herniated canine nucleus pulposus material as loading control.


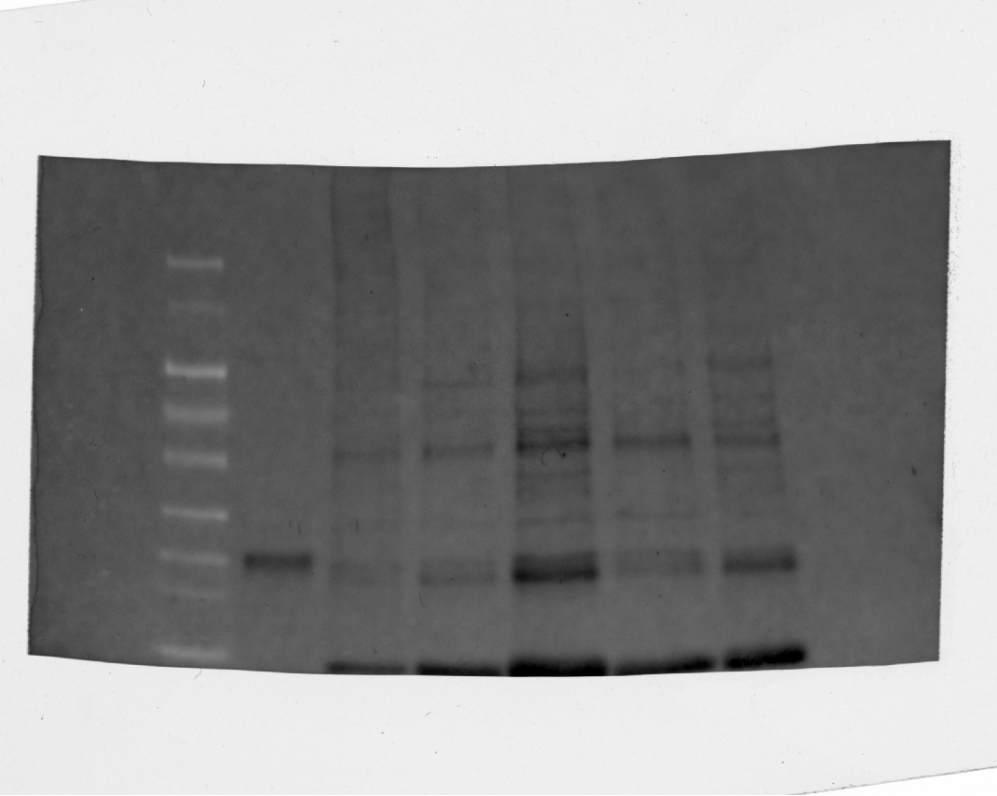
(A)
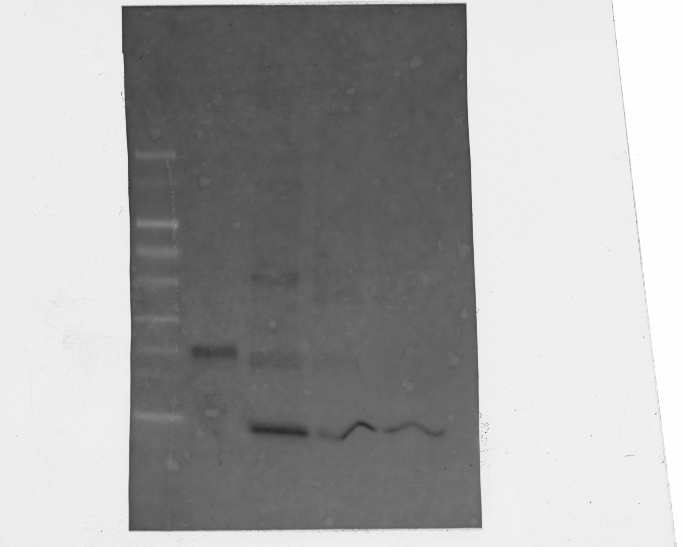
(B)

**Legend supplementary Figure 1:** **Stain free blot of herniated A) and non-herniated B) canine nucleus pulposus material. (**A) Lane 1: 1.5 μl Page Ruler^TM^ Prestained Protein Ladder (26616, Thermo Scientific), Lane 2: 1x10^-4 µg, 30 kDa proteolytic fragments from human plasma fibronectin (F9911, Sigma-Aldrich); Lane 3: Patient 1; Lane 4: Patient 2; Lane 5: Patient 3; Lane 6: Patient 4; Lane 7, Patient 5; (B) Lane 1: 1.5 μl Page Ruler^TM^ Prestained Protein Ladder, Lane 2: 1x10^-4 µg, 30 kDa proteolytic fragments from human plasma fibronectin (F9911, Sigma-Aldrich); Lane 3: Patient 4; Lane 4: Patient 6; Lane 5: Patient 7; (for patient description see Table 1)

# Supplementary Figure 2: Unprocessed western blot of herniated and non-herniated canine nucleus pulposus material.


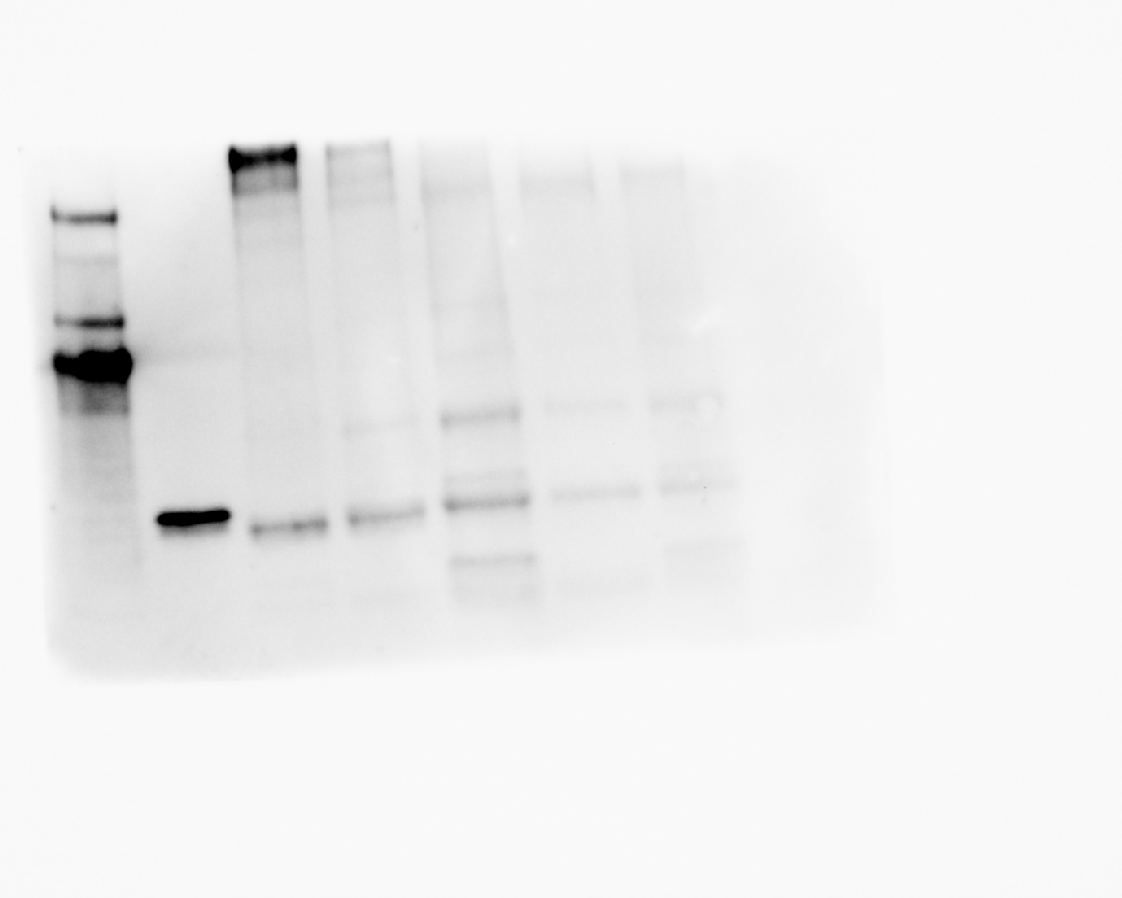
(A)**
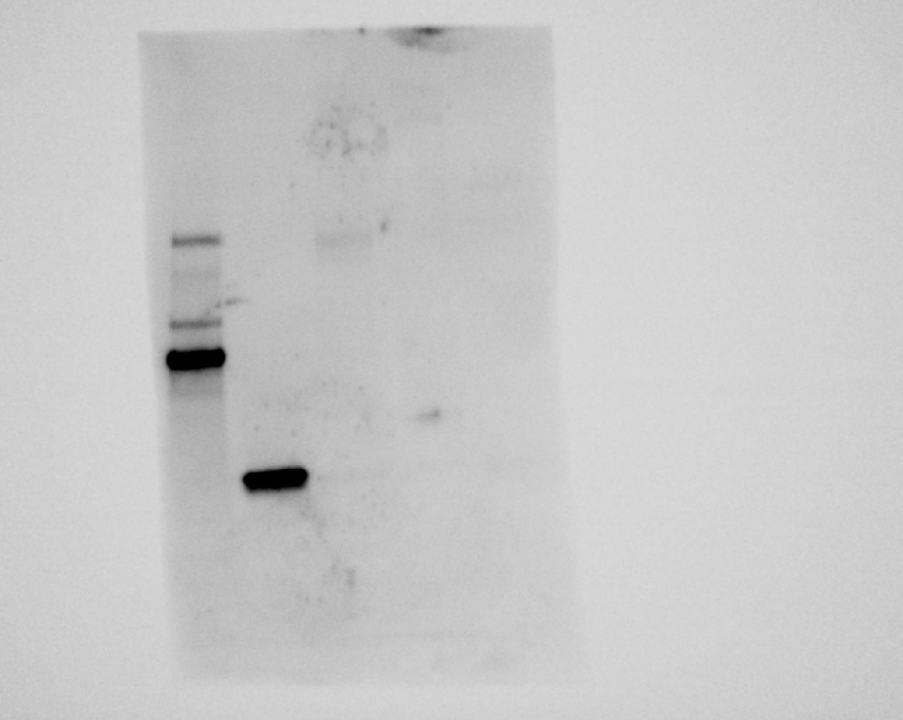
**(B)

**Legend supplementary Figure 2: Western blot of herniated and non-herniated canine nucleus pulposus material.** Fn-f were detected using a mouse monoclonal antibody specific for the N-terminal end of fibronectin (7D5, Prof. Deane Mosher, University of Wisconsin, Madison, WI, USA). (A) Lane 1: 1.5 μl Page Ruler^TM^ Prestained Protein Ladder (26616, Thermo Scientific), Lane 2: 1x10^-4 µg, 30 kDa proteolytic fragments from human plasma fibronectin (F9911, Sigma-Aldrich); Lane 3: Patient 1; Lane 4: Patient 2; Lane 5: Patient 3; Lane 6: Patient 4; Lane 7, Patient 5; (B) Lane 1: 1.5 μl Page Ruler^TM^ Prestained Protein Ladder; Lane 2: 1x10^-4 µg, 30 kDa proteolytic fragments from human plasma fibronectin (F9911, Sigma-Aldrich); Lane 3: Patient 4; Lane 4: Patient 6; Lane 5: Patient 7; (for patient description see Table 1)

# Supplementary Figure 3: IL-6 and PGE_2_ secretion of NP cells after exposure to 30 kDa fibronectin fragments alone and in combination with Sparstolonin B and IL-1β.

#
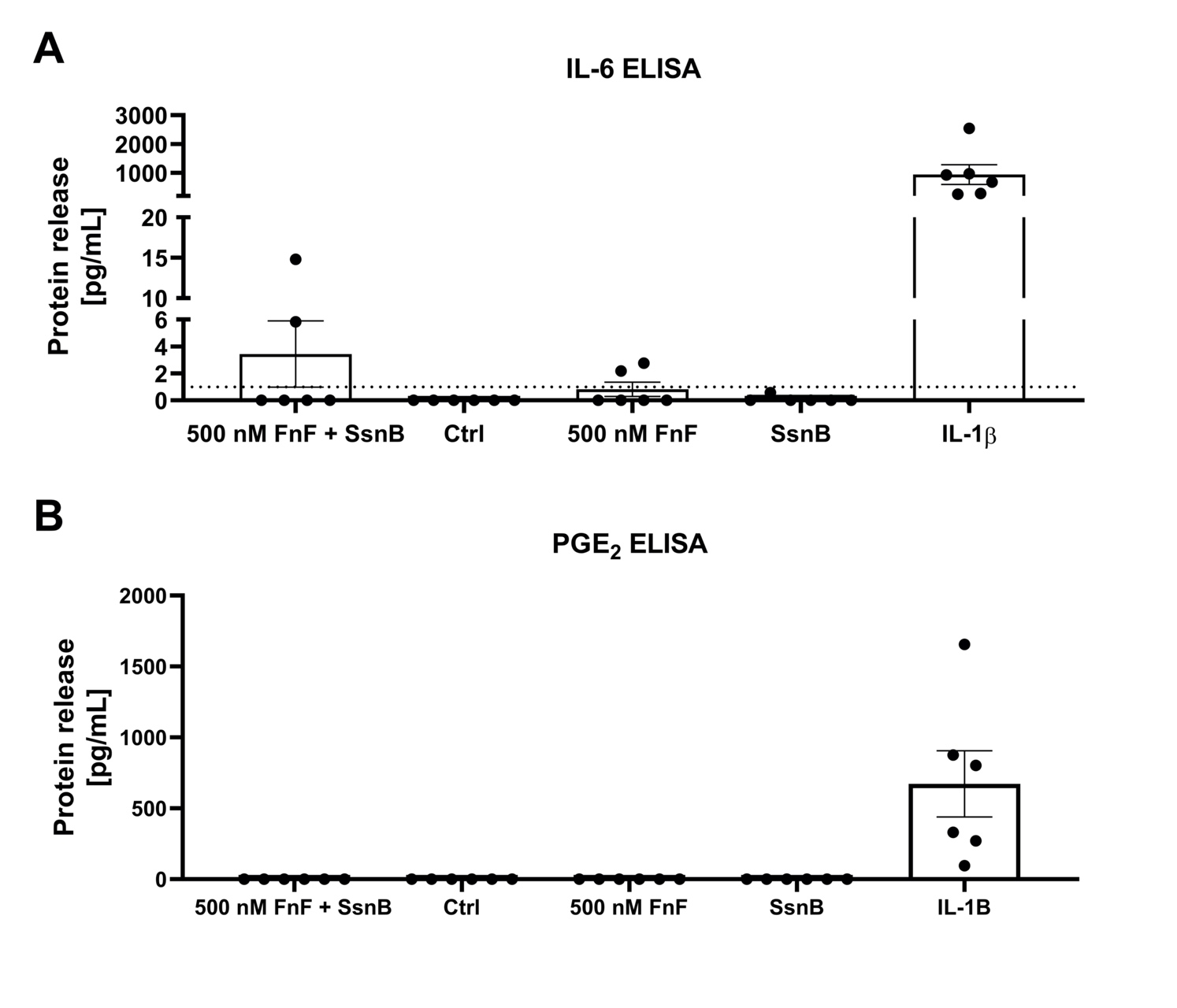


**Legend supplementary Figure 3: IL-6 ELISA (A) and PGE_2_ (B) ELISA of NP cell culture supernatant.** IL-6 (A) and PGE_2_ (B) release of NP cells without treatment (Ctrl = controls) and after 18h exposure to 30kDa fibronectin fragments (Fn-f; 500nM), IL-1β, 30kDa Fn-f with the Toll-Like-Receptor inhibitor Sparstolonin B (SsnB) and Sparstolonin B alone. Protein concentrations secreted into the cell culture supernatant by NP cells were measured by ELISA and presented as pg/ mL on the y-axis.
